# Application of Systems Biology and Bioinformatics Methods in Biochemistry and Biomedicine 2014

**DOI:** 10.1155/2015/568607

**Published:** 2015-04-19

**Authors:** Yudong Cai, Tao Huang, Lei Chen, Bing Niu

**Affiliations:** ^1^College of Life Sceince, Shanghai University, Shanghai 200444, China; ^2^Department of Genetics and Genomics Sciences, Mount Sinai School of Medicine, New York, NY 10029, USA; ^3^College of Information Engineering, Shanghai Maritime University, Shanghai 201306, China; ^4^College of Life Science, Shanghai University, Shanghai 200444, China

In biochemistry and biomedicine, more and more new technologies are developed and the high-throughput data generated by such technologies need to be analyzed with more powerful systems biology and bioinformatics methods.

In this special issue, novel systems biology and bioinformatics methods developed in 2014 and their applications in biochemistry and biomedicine were introduced.

Ł. Palkowski et al. presented a SAR study of a novel homologous series of bis-quaternary imidazolium chlorides. With the help of the dominance-based rough set approach (DRSA), some relevant features were extracted, which were deemed to be highly related to antifungal activity of the compounds.

H. Liu et al. developed a new method to predict HIV-1 protease cleavage site. They used two feature fusion methods: combination fusion and decision fusion and improved the prediction performance. Their results and analysis provide useful instruction and help for designing HIV-1 protease inhibitor in the future.

G. P. Monterrubio-López et al. identified novel potential vaccine candidates against tuberculosis based on reverse vaccinology. For each candidate, both comprehensive literature survey and bioinformatics analysis, such as the simulation of the immune response, were conducted. At last, six novel vaccine candidates, EsxL, PE26, PPE65, PE_PGRS49, PBP1, and Erp, were considered to be useful for tuberculosis vaccine design.

M. Aqil et al. analyzed the transcriptome of human monocytic cells expressing the HIV-1 Nef protein and their exosomes. They identified four key mRNAs, MECP2, HMOX1, AARSD1, and ATF2, which are important for chromatin modification and gene expression. They also identified three key mRNAs, AATK, SLC27A1, and CDKAL, which are important in apoptosis and fatty acid transport.

Y. Liu et al. developed a computational method to predict protein glycation sites by using the support vector machine classifier, maximum relevancy minimum redundancy (mRMR), and incremental feature selection (IFS) method. Their prediction accuracy was 85.51% and MCC (Matthews correlation coefficient) was 0.70. They found that the composition of k-spaced amino acid pairs feature contributed the most for glycation sites prediction.

Z. Xu et al. proposed a systems biology approach to quantify biofilm formation of* P. aeruginosa* upon the changes of availability of amino acids, ferrous ions, sulfate, and phosphate in the surrounding environment. Some biofilm formation patterns were discovered, which can be validated by existing experimental data.

F. Wang et al. presented a distribution-based approach for gene pair classification by identifying a disease-specific cutoff point that classified the coexpressed gene pairs into strong and weak coexpression structures. They applied their method to analyze the NPM1-associated genes in chronic myelogenous leukemia (CML) and found that genes involved in the ribosomal synthesis and translation process tended to be coexpressed in the CML group.

Y. Cui et al. collected the biochemical examination and tongue image data from 46 case subjects with hyperuricemia and 46 control subjects. Based on the symmetrical Haar-like features which were extracted from tongue images, they built a classifier that can distinguish the case and control subjects. The area under the receiver operating characteristic curve (AUC) was 0.877.

M.-H. Wang and W.-H. Chang investigated the effects of electrode geometry in microfluidic devices on the impedance of single HeLa cell. Their simulations indicated that the circle and parallel electrodes provide higher electric field strength compared to cross and standard electrodes at the same operating voltage. Increasing the operating voltage reduces the impedance magnitude of a single HeLa cell in all electrode shapes and decreasing impedance magnitude of the single HeLa cell increases measurement sensitivity.

M. Deng et al. applied gas chromatography-mass spectrometry (GS-MS) in combination with multivariate statistical analysis to explore the metabolic variability in urine of chronically hydrogen sulfide- (H_2_S-) poisoned rats relative to control ones. Their technique can be employed to decipher the mechanism of chronic H_2_S poisoning and promote the use of metabolomics in clinical toxicology.

W. Hu et al. evaluated the accuracy of a novel computer-simulated biopsy marking system (CSBMS) developed for endoscopic marking of gastric lesions. Twenty-five patients with history of gastric intestinal metaplasia received both CSBMS-guided marking and India ink injection in five points in the stomach at index endoscopy. The mean accuracy of CSBMS at angularis was 5.3 ± 2.2 mm, antral lesser curvature 5.7 ± 1.4 mm, antral greater curvature 6.1 ± 1.1 mm, antral anterior wall 6.9 ± 1.6 mm, and antral posterior wall 6.9 ± 1.6 mm. Their results suggested that the CSBMS can accurately identify previously marked gastric sites by endoscopic tattooing within 1 cm on follow-up endoscopy.

G. Huang et al. developed a method to predict S-nitrosylation modification sites based on kernel sparse representation classification and mRMR Algorithm. Their predictor achieves Matthews' correlation coefficients of 0.1634 and 0.2919 for the training set and the testing set, respectively, which are better than those of *k*-nearest neighbor algorithm, random forest algorithm, and sparse representation classification algorithm. A webserver for the prediction of S-nitrosylation sites based on kernel sparse representation classification and minimum Redundancy Maximum Relevance algorithm is available at http://www.zhni.net/snopred/index.html.

*Yudong Cai* *Tao Huang* *Lei Chen* *Bing Niu*

